# Clinical improvement of premature ventricular contraction in an elderly patient treated with Chaihu Sanshen Capsule: A case report

**DOI:** 10.1097/MD.0000000000050641

**Published:** 2026-09-18

**Authors:** Tao He, Zhuhui Zhang, Jianhe Liu

**Affiliations:** a Department of Cardiovascular, The First Hospital of Hunan University of Chinese Medicine, Changsha, Hunan, China.

**Keywords:** case report, Chaihu Sanshen Capsule, elderly, premature ventricular contraction, traditional Chinese medicine

## Abstract

**Rationale:**

Premature ventricular contraction (PVC) is one of the most common kinds of arrhythmias for which the treatment poses a therapeutic dilemma. Traditional Chinese medicine has a long history in arrhythmia treatment, and this is a rare case of an elderly patient with PVC who showed significant improvement after receiving Traditional Chinese medicine treatment.

**Patient concerns:**

An 82-year-old male, with a history of mitral stenosis, has undergone surgical mitral valve replacement. This time, he presented with chest pain and palpitations. He was diagnosed with frequent PVCs.

**Diagnoses:**

He was diagnosed with PVC by 24-hour Holter monitoring.

**Interventions:**

The patient visited The First Hospital of Hunan University of Chinese Medicine on November 4, 2024, and received therapy with Chaihu Sanshen Capsule (CHSSC).

**Outcomes:**

After taking CHSSC for 3 treatment courses, the Holter tests showed that the number of ectopic premature beats decreased from 47,730 to 844 within 24 hours. This objective improvement was correlated with significant symptomatic relief, including a pronounced reduction in palpitations and easing of chest tightness.

**Lessons:**

This single case observation generates the hypothesis that CHSSC may be associated with reduced PVC burden and symptomatic improvement in elderly patients. Nevertheless, these preliminary findings warrant further investigation through larger prospective studies to establish conclusive evidence.

## 1. Introduction

Premature ventricular contractions (PVCs) are ectopic impulses originating from the ventricles, typically manifesting on the electrocardiogram (ECG) as wide QRS complexes (usually > 120 ms) not preceded by P waves. The morphological features of these ectopic beats can provide important clues for localizing their anatomical site of origin within the ventricles.^[1]^

PVC is one of the most frequently observed arrhythmias in clinical practice, with an increasing incidence in recent years. The detected prevalence is highly dependent on the diagnostic methodology. Conventional electrocardiography detects PVC in about 1to 4% of the general population, whereas 24 to 48 hours ambulatory ECG monitoring reveals a markedly higher prevalence of up to 75%.^[2–4]^ This arrhythmia can be found across a diverse population, from those with underlying cardiovascular diseases to clinically healthy individuals.

The primary etiological factors of PVC encompass myocardial injury (e.g., ischemia, hypoxia, myocarditis, or cardiac surgery), drug-induced toxicity (e.g., from digitalis and quinidine), and lifestyle triggers including emotional stress and electrolyte disturbances.^[5]^ Chronic, high-burden PVCs are associated with an increased risk of cardiomyopathy, malignant arrhythmias, and sudden cardiac death, posing a major threat to human health.^[6,7]^

Current management focuses on symptom control and prognostic improvement. When pharmacotherapy with beta-blockers or amiodarone fails, radiofrequency ablation is considered, yet its success is highly location-dependent and costly.^[8–10]^ Crucially, in the presence of structural heart disease, which is a common scenario in elderly patients, the priority shifts to managing the underlying pathology, as antiarrhythmic drugs carry increased risks of pro-arrhythmia and heart failure exacerbation. This underscores a critical therapeutic gap for this vulnerable population.

Traditional Chinese Medicine (TCM) offers a complementary strategy. Formulations like Shensong Yanxin Capsule have shown efficacy in reducing PVC burden, primarily through ion channel modulation.^[11–14]^ However, evidence for TCM formulas acting through a paradigm of soothing the liver and regulating qi, a concept rooted in the theory that liver qi stagnation gives rise to blood stasis and cardiac discomfort, remains scarce. Chaihu Sanshen Capsule (CHSSC), a compound of Bupleurum Cinense, Panax notoginseng, and Radix Salviae Miltiorrhizae, operates through this distinct mechanistic paradigm. Unlike ion channel-targeting drugs, recent research indicated it may suppress ventricular arrhythmia by inhibiting the CaMKII/FKBP12.6/RyR2/Ca2 + signaling pathway and mitigating myocardial injury, offering a fundamentally different therapeutic approach.^[15]^

The primary challenges in treating this patient are twofold. First, the patient’s advanced age complicates drug selection and increases vulnerability to adverse effects of conventional antiarrhythmics. Second, the presence of structural heart disease (status post-mitral valve replacement) limits the safety profile of many standard therapies. Despite these challenges, there is a marked absence of literature documenting the effects of liver-soothing, blood-activating TCM formulas in elderly patients with structural heart disease. This case report aims to fill this gap by detailing, for the first time, the clinical response to CHSSC in an elderly patient with frequent PVCs following cardiac surgery, thereby generating hypotheses for future investigation.

## 2. Case presentation

An 82-year-old male patient presented to our hospital on November 4, 2024, with recurrent chest pain over 4 years that had significantly worsened in the preceding 4 days. His medical history included well-controlled Grade 3 hypertension and prior mitral valve surgery. He denied smoking, alcohol use, or other comorbidities.

**Figure 1.**
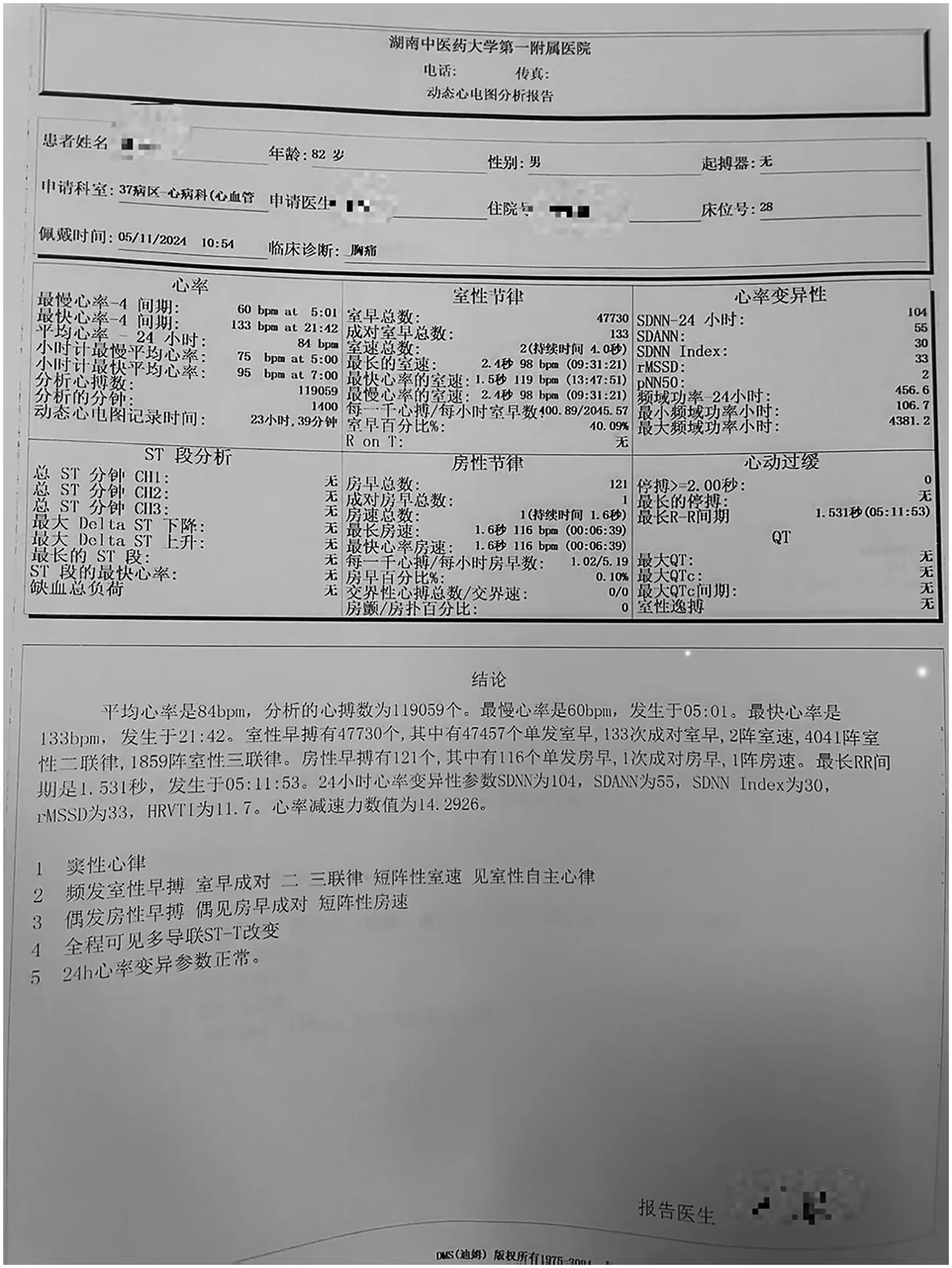
24-hour Holter monitoring revealed a total of 119,059 heartbeats and 47,730 PVCs in the patient before taking CHSSC. CHSSC = Chaihu Sanshen Capsule, PVCs = premature ventricular contractions.

In 2021, the patient had undergone transcatheter aortic valve implantation at the Second Xiangya Hospital, Central South University, for severe aortic stenosis. He was maintained on warfarin postoperatively and reported regular adherence. At current presentation, the patient reported exertional chest pain, palpitations, diaphoresis, xerostomia with bitter taste, and fatigue. Electrocardiography revealed ventricular premature beats, and 24-hour ambulatory ECG monitoring confirmed frequent PVCs (Fig. 1). Echocardiography showed no notable structural abnormalities beyond the known valvular status. The treating physician proposed catheter ablation, which the patient declined, opting for pharmacological management. Secondary causes of PVCs, including thyroid dysfunction and electrolyte disturbances, were excluded. The patient was started on CHSSC at a dosage of 4 capsules 3 times daily, taken 30 minutes after meals. Throughout the treatment period, hepatic and renal function remained stable, and no significant adverse events were observed. The patient reported substantial improvement in palpitations and partial resolution of chest tightness, and was subsequently discharged for outpatient follow-up.

**Figure 2.**
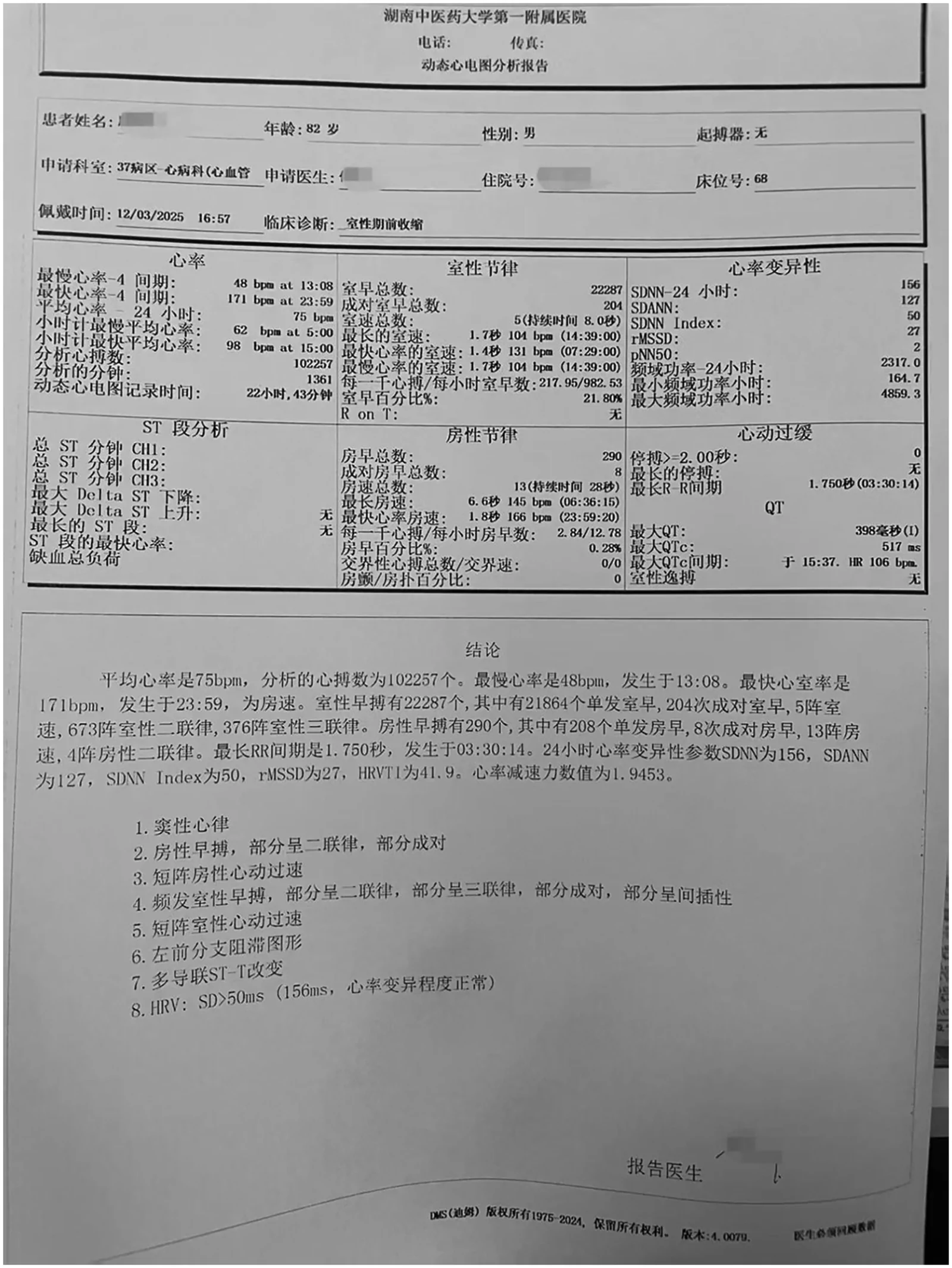
24-hour Holter monitoring revealed a total of 102,257 heartbeats and 22,287 PVCs in the patient after taking CHSSC for 4 months. CHSSC = Chaihu Sanshen Capsule, PVCs = premature ventricular contractions.

At the follow-up in March 2025, ambulatory ECG monitoring showed a marked reduction in PVC burden compared to baseline, correlating with the patient’s clinical improvement (Fig. 2). No symptomatic hypotension or bradycardia was documented. The treatment regimen was to be continued.

**Figure 3.**
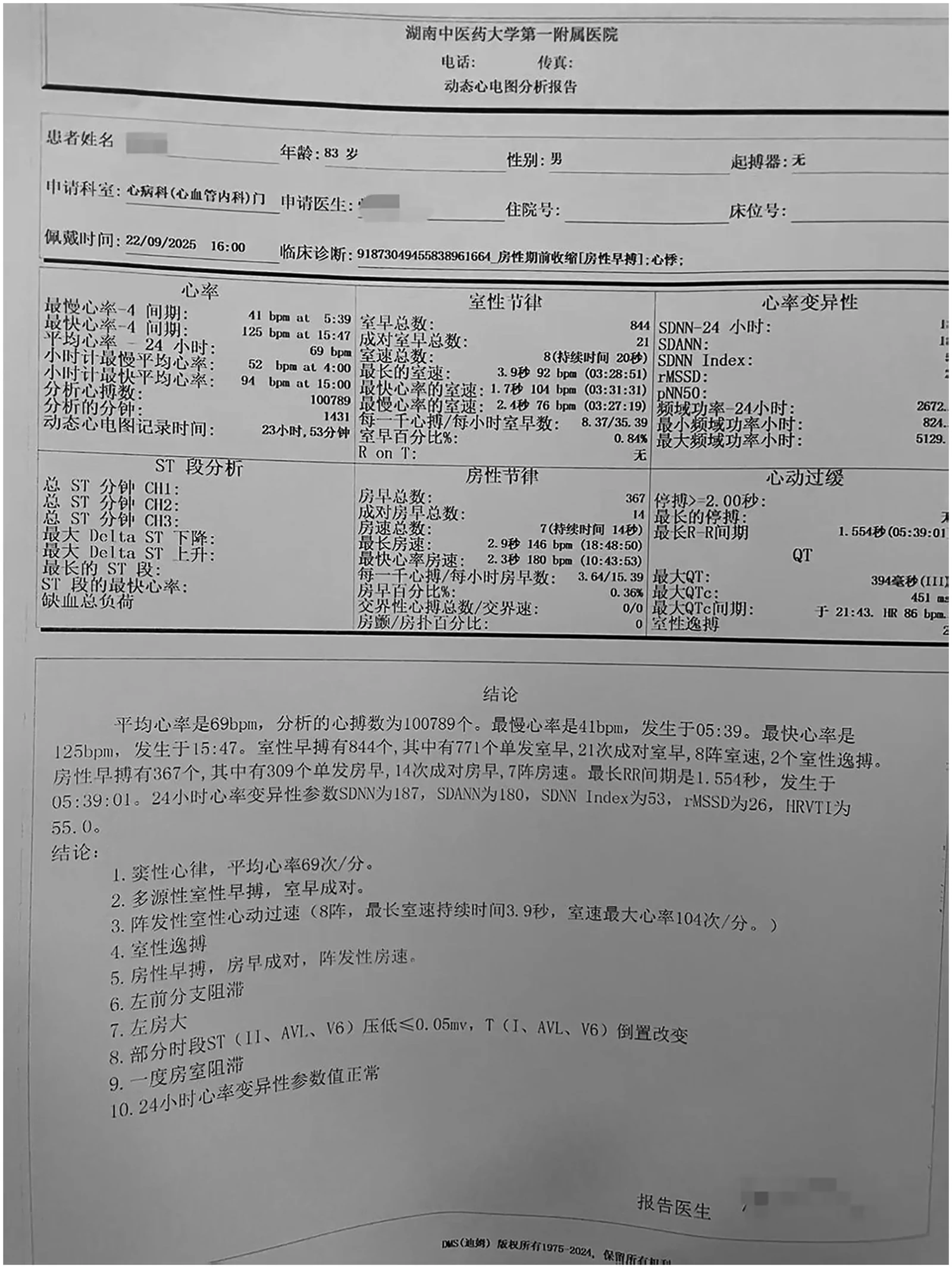
24-hour Holter monitoring revealed a total of 100,789 heartbeats and 844 PVCs in the patient after taking CHSSC for 9 months. CHSSC = Chaihu Sanshen Capsule, PVCs = premature ventricular contractions.

**Figure 4.**
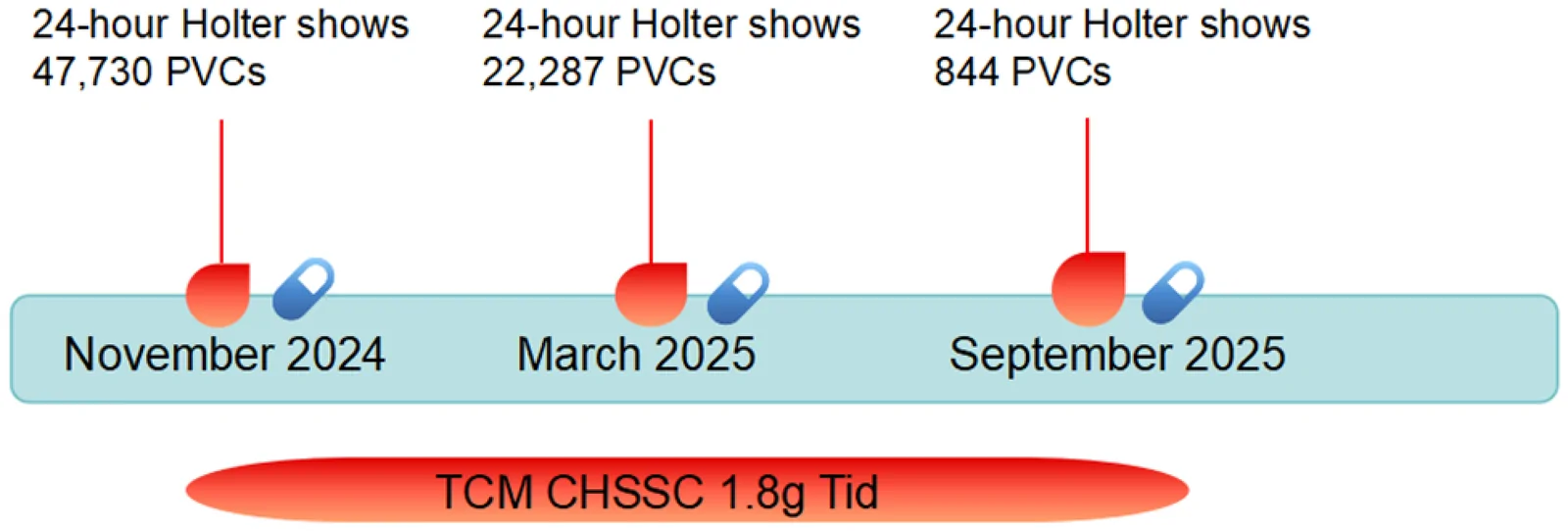
Timeline of symptom changes in the process of taking CHSSC. CHSSC = Chaihu Sanshen Capsule, PVCs = premature ventricular contractions, TCM = Traditional Chinese medicine.

At a subsequent follow-up in September 2025, repeated 24-hour Holter monitoring documented a total of 100,789 heartbeats with 844 PVCs, reducing from the initial burden of 47,730 PVCs per 24 hours (Fig. 3). The patient remained largely asymptomatic despite the persistent PVCs. A timeline chart has been included to depict the relationship between the sequential phases of CHSSC therapy and the evolving PVC symptom profile (Fig. 4).

## 3. Discussion

From the perspective of TCM, the modern diagnosis of PVCs corresponds to the classical categories of “palpitations” and “anxiety” based on shared symptomatic profiles. The core pathogenesis involves the heart and typically follows a pattern of deficiency in its root and excess in its manifestations. The syndrome of dual deficiency of qi and yin is identified as a principal pattern, in which the heart becomes deprived of nourishment. Over time, this progresses to damage yin and blood, causing the heart vessels to lose their capacity for moistening and nourishment, directly generating symptoms such as palpitations, restlessness, shortness of breath, and fatigue. If the qi and yin deficiency persists long-term, it can lead to spleen dysfunction and internal production of phlegm-heat.^[16]^ The ascent of this phlegm-fire to harass the heart spirit is a key mechanism in the onset and persistence of palpitations.

CHSSC is a proprietary formulation developed by the First Affiliated Hospital of Hunan University of Chinese Medicine, with a history of clinical use spanning many years. This compound preparation is derived from the classical formula Xiao Chaihu Decoction, documented in the “*Treatise on Febrile Diseases*” and is formulated based on TCM theory, with the primary actions of moving qi, activating blood circulation, and resolving phlegm. Its complete composition is detailed in Table 1. The capsules have demonstrated notable clinical efficacy in managing arrhythmias in prior observational studies within our center.

**Table 1:** The composition of Chaihu Sanshen capsule (CHSSC).

Chinese name	Latin name	Drug proportion
Chai Hu	Bupleuri Radix	15
Huang Lian	Rhizoma Coptidis	6
Dan Shen	Salvia miltiorrhiza Bge	10
Fa Ban Xia	Pinellia ternata (Thunb.) Makino	10
Ku Shen	Sophora flavescens Ait	10
Qing Hao	Artemisia annua L	10
Dang Shen	Codonopsis pilosula (Franch.) Nannf	15
Gan Cao	Glycyrrhiza uralensis Fisch	6

Mechanistically, within the formula, Bupleuri Radix (Chai Hu) serves to regulate qi and harmonize the Shaoyang system. It is combined with Coptidis Rhizoma (Huang Lian), which clears and purges Shaoyang heat, thereby assisting in the reconciliation process. Codonopsis Radix (Dang Shen) strengthens the spleen, tonifies the middle jiao, and generates body fluids, providing a source for qi and facilitating its smooth movement by Bupleurum. Salviae Miltiorrhizae Radix (Dan Shen) promotes blood circulation, removes blood stasis, clears the mind, and relieves irritability. As blood is the mother of qi, its activation by Salvia helps carry qi throughout the body. The combination of Bupleurum and Salvia integrates qi regulation with blood activation, facilitating the circulation of both. Sophorae Flavescentis Radix (Ku Shen) clears heat and dries dampness, while Pinelliae Rhizoma (Ban Xia), specially processed, excels at drying dampness, resolving phlegm, and dispersing nodules. Artemisiae Annua Herba (Qing Hao) resolves phlegm and alleviates palpitations. These latter herbs act as important adjuvants. Finally, Glycyrrhizae Radix (Gan Cao) harmonizes the middle energizer and moderates the effects of all other components in the formula. Collectively, the formula works synergistically to relieve palpitations, tonify qi, activate blood, clear heat, and resolve phlegm.

Accumulating evidence from modern pharmacological research indicates that multiple active components in CHSSC contribute to its antiarrhythmic properties in experimental models. Berberine, an active alkaloid from Coptidis Rhizoma (Huang Lian), protects cardiomyocytes by inhibiting calcium overload and suppressing triggered activity, thereby counteracting arrhythmogenesis.^[17]^ Furthermore, berberine inhibits the transformation of cardiac fibroblasts into myofibroblasts, which helps suppress sympathetic remodeling and reduces arrhythmia susceptibility.^[18]^ The alkaloids derived from Pinelliae Rhizoma (Ban Xia) exhibit quinidine-like antiarrhythmic activity. In Glycyrrhizae Radix (Gan Cao), both glycyrrhizin and isoliquiritin have been identified as active antiarrhythmic constituents. Glycyrrhizin blocks the Nav1.5 channel, while isoliquiritin acts as a dual blocker of the hERG and Nav1.5 channels, collectively contributing to Class I and III antiarrhythmic effects.^[19]^ Oxymatrine, a key compound from Sophorae Flavescentis Radix (Ku Shen), effectively prolongs the QT interval and the effective refractory period, raises the diastolic excitation threshold, and reduces myocardial automaticity and triggered activity.^[20]^ Additionally, artemisinin derived from Artemisiae Annua Herba (Qing Hao) may exert antiarrhythmic effects through inhibition of potassium channels, including the transient outward potassium current.^[21]^ These preclinical findings offer a plausible mechanistic context that may help explain the favorable clinical trajectory observed in this patient. However, they do not constitute direct evidence of the formulations clinical efficacy in this case.

Our prior research has established that CHSSC protects against myocardial ischemia-reperfusion injury via the PI3K/AKT/p53 pathway and PKCβ II/NOX2/ROS signaling pathway.^[22,23]^ In addition to this protective role, we further elucidated that CHSSC effectively attenuates intracellular calcium overload following ischemic insult, thereby ameliorating the underlying pro-arrhythmic substrate and reducing the incidence of resultant ventricular arrhythmias.^[15]^ These experimental observations further support the biological plausibility of a potential antiarrhythmic effect, though their translational relevance to the present clinical scenario remains to be established.

Several important limitations of this case report must be acknowledged. First and foremost, causality cannot be determined from a single observational case. As a single-case observation without a control group, this report cannot distinguish treatment effects from spontaneous fluctuation or regression to the mean. Second, the patient’s advanced age (82 years), underlying structural heart disease (status post-mitral valve replacement and), and single-center setting restrict the generalizability of these findings to broader PVC populations. Third, the patient’s medication adherence and potential lifestyle or psychological confounders were not systematically quantified, which may have influenced symptom perception and clinical outcomes. Finally, the follow-up duration, while extending to September 2025, remains insufficient to assess the long-term sustainability of the observed response or its impact on hard clinical endpoints such as mortality or major adverse cardiovascular events.

In summary, this case provides preliminary, hypothesis-generating observations that treatment with CHSSC was associated with symptomatic relief and a substantial reduction in PVC burden in an elderly patient with frequent PVCs following cardiac surgery. These observations enrich the clinical literature on TCM for PVC and suggest a potential role for this formulation in integrative cardiology that warrants further investigation. However, given the inherent limitations of a single case report, particularly the absence of a control group and the inability to establish causality from a single observation, no definitive conclusions regarding clinical efficacy can be drawn from this report. Larger prospective, controlled studies with longer follow-up periods are urgently needed to verify the safety and effectiveness of CHSSC and to establish its definitive role in evidence-based treatment algorithms for PVC.

## Acknowledgments

We are also grateful to all the researchers, including the physicians, nurses, and technicians, who participated in this study.

## Author contributions

**Conceptualization:** Tao He, Zhuhui Zhang.

**Formal analysis:** Tao He, Zhuhui Zhang, Jianhe Liu.

**Investigation:** Tao He, Jianhe Liu.

**Validation:** Tao He, Jianhe Liu.

**Visualization:** Tao He, Jianhe Liu.

**Data curation:** Zhuhui Zhang.

**Funding acquisition:** Jianhe Liu.

**Writing – original draft:** Tao He, Zhuhui Zhang.

**Writing – review & editing:** Tao He, Jianhe Liu.
